# The incidence of non-affective psychotic disorders in low and middle-income countries: a systematic review and meta-analysis

**DOI:** 10.1007/s00127-022-02397-6

**Published:** 2022-12-22

**Authors:** Rayanne John-Baptiste Bastien, Tao Ding, Alfonso Gonzalez-Valderrama, Lucia Valmaggia, James B. Kirkbride, Hannah E. Jongsma

**Affiliations:** 1 PsyLife Group, Division of Psychiatry, UCL, London, UK; 2 Department of Statistical Sciences, UCL, London, UK; 3 Early Intervention Program, Instituto Psiquiátrico Dr J Horwitz Barak, Santiago, Chile; 4 School of Medicine, Universidad Finis Terrae, Santiago, Chile; 5 Department of Psychology, Institute of Psychiatry, Psychology and Neuroscience, London, UK; 6 South London and Maudsley NHS Trust, London, UK; 7 Clinical Psychiatry, KU Louvain, Louvain, Belgium; 8 Centre for Transcultural Psychiatry ‘Veldzicht’, Balkbrug, The Netherlands; 9 University Centre for Psychiatry, University Medical Centre Groningen, Groningen, The Netherlands

**Keywords:** Psychotic disorders, Global mental health, Systematic review, LMIC, Global south, Incidence, Epidemiology, Schizophrenia

## Abstract

**Purpose:**

Global understanding of the epidemiological landscape of non-affective psychotic disorders (NAPD) is predominantly based on studies from high-income countries. We sought to systematically review and meta-analyse all incidence studies conducted in low and middle-income countries (LMICs).

**Methods:**

We systematically searched four databases using terms for NAPD, incidence and LMICs. Citations were eligible for inclusion if: published between 1 January 1960 and 31 May 2022; wholly or partially conducted in an LMIC, and; containing data on NAPD incidence in the general adult population. Two independent raters assessed study quality according to previously published criteria. We conducted a narrative synthesis and random-effects meta-analyses where sufficient studies were available (*N* ≥ 5).

**Results:**

We retrieved 11 421 records, of which 23 citations met inclusion criteria from 18 unique studies across 19 settings in 10 LMICs. Median study quality was 4 out of 7 (interquartile range: 3–6). The crude incidence of NAPD varied around 4.2 times, from 10.0 per 100,000 person-years (95% confidence interval [CI] 8.7–11.4) in Brazil to 42.0 (95%CI 32.2–54.8) in India, with marked heterogeneity in methodologies and rates. Our 60-year review highlights the dearth of robust evidence on the incidence of psychotic disorders in LMICs.

**Conclusion:**

Without reliable, contemporary estimates of this fundamental cornerstone of population health, it is impossible to understand the true burden, distribution or causes of psychotic disorders in over 87% of the world’s population. A new, more equitable global mental health evidence base for NAPD is now urgently required.

**Supplementary Information:**

The online version contains supplementary material available at 10.1007/s00127-022-02397-6.

## Introduction

In little over 15 years, received wisdom on the epidemiology of psychotic disorders has changed from the view that they were uniformly distributed, to current understanding of a robust, replicable but varied distribution by person and place [1]. For example, systematic reviews and meta-analyses have confirmed that incidence rates of psychotic disorders are higher for younger men than women, peaking in late adolescence, before declining throughout adult life [2–4]. Rates are also consistently elevated for many ethnic minority groups in most settings where this has been studied [2–5], and appear higher in those exposed to more urban environments during the life course [6]. These findings potentially inform both aetiological understanding of psychotic disorders and the provision of timely and appropriate early intervention for psychosis.

Nonetheless, the epidemiological evidence base on which these findings are predicated arises almost exclusively from high-income countries [HICs], with only occasional, older exceptions [7–10]. This is problematic for several reasons: first, observed patterns of the distribution of psychotic disorders in one country or narrow range of countries may not generalise to other settings; second, alternate patterns of exposures and outcomes in other settings may refute, support or refine current aetiological understanding, and; finally, epistemological thinking restricted to a small set of countries may create hegemonic structures which fail to reveal the true underlying aetiology of psychotic disorders across different settings. In other words: what we think we know about non-affective psychotic disorders [NAPD] is mainly based on evidence from HICs, and this might not hold true universally.

A useful example is the study of the association between urbanicity and psychosis. Studies conducted in HICs frequently report higher incidence rates of NAPD in more urban populations [6], including associations with urban birth [11]. However, results from a cross-sectional survey of 42 Low and Middle Income Countries [LMICs] in the World Health Organization [WHO] World Health Survey [12], reported no consistent association between the prevalence of psychotic symptoms and contemporaneously-estimated rural or urban exposure. Although comparing such findings is difficult [13], recent evidence from a nationwide study in Chile [14] and multinational [15] incidence data from Europe and Brazil also found that regional deprivation was more strongly associated with the incidence of psychotic disorders than population density, usually considered a more direct marker of urbanicity; these studies suggest that different patterns of association between risk factors and psychosis may be present in different contexts. Unfortunately, evidence on the incidence of psychotic disorders—a central epidemiological cornerstone for understanding the burden, distribution, and aetiology of disease—from LMICs is scant. To address and quantify this issue, our objective was to systematically review the literature on the incidence of NAPDs in LMICs published between January 1960 and May 2022.

## Methods

### Search strategy and selection criteria

In this systematic review and meta-analysis we followed PRISMA guidelines (Supplemental Table 1), including protocol preregistration on PROSPERO (reference: CRD42020179678). We adapted a previous methodology we developed for global and national systematic reviews [2, 4], based on Cochrane systematic reviewing guidelines. Briefly, we systematically searched MedLine, PsycINFO, Web of Science, and Embase using a comprehensive search strategy (Supplemental Materials, Sect. 1) with application of the Cochrane filter for LMICs [16]. Citations were eligible for inclusion if they were:

- published 1 January 1960—31 May 2022;
- wholly or partially conducted in a LMIC (as defined by The World Bank [17]);
- contained sufficient original data on, or to derive, the incidence of NAPD;
- conducted in the general adult population (aged ~ 16–64).

Citations published between 1960 and 2001 and 2019–2022 were retrieved by searching the aforementioned databases. Citations published between 2002 and 2018 were identified from an existing open access database maintained by HEJ from our recent global review of the literature using an identical search strategy [4]. We also searched the references of included citations for potentially missed citations, as well as the resource databank of the Global Burden of Disease Study [18]. We placed no restriction on language of publication, study design or publication status, although grey literature was only identified via conference proceedings, author correspondence, and reference searching. One author (RJ-BB) carried out the searches, and three authors (RJ-BB, JBK, HEJ) screened citations at title stage. Two authors (HEJ, RJ-BB) independently and in duplicate screened citations at abstract and full-text review stages (Supplemental Materials, Sect. 2), with disagreements solved by consensus with JBK.

### Data extraction

One author extracted data from included studies (RJ-BB), with consistency and accuracy checks performed by HEJ and JBK. Rate-level data about incidence, and meta-level data on study characteristics, time period and study quality were included.

Our primary outcome was the crude incidence of NAPD per 100,000 person-years, based on the diagnostic classification system used in each study (Supplemental Materials, Sect. 3). Under the International Classification of Diseases (10th revision) [ICD-10], this was defined as F20–29 and includes schizophrenia, schizotypal disorder, persistent delusional disorder, acute and transient psychotic disorders, induced psychotic disorders, schizoaffective disorders, other non-organic psychotic disorders and unspecified non-organic psychosis. We assumed sufficiently commonality across systems to permit comparison of rates. Though this was not an inclusion criterion, here available, we also extracted and reported incidence rates for: schizophrenia (F20); affective psychoses (F30.2, F31.2, F32.3, F33.3), and; all clinically relevant psychotic disorders (F20-33, F1X.5). We only included studies where a formal diagnosis of NAPD was made, so religious, alternative or traditional healers were only included if there was a cooperation with a medical care provider. We recorded the numerator (case) and denominator (population at-risk), rate, standard error and 95% confidence intervals [95%CI], where reported. Where available, we extracted incidence data by sex.

We also recorded meta-level data on study design, study quality, and time period. Study design was defined as first contact (with any healthcare provider for NAPD), first episode (of NAPD), first admission (to any psychiatric facility for NAPD), cohort or household surveys. Two independent raters (AG-V and LV) assessed study quality according to seven previously published criteria relevant to incidence studies of psychotic disorders [2, 4] (Supplemental Materials, Sect. 4). Discrepancies were resolved by consensus with HEJ and JBK. Time period was defined as the median year of the case ascertainment period. Where multiple citations reported data from the same sample, both were included in the systematic review, with a core citation defined based on the one containing the most detailed estimates of incidence for inclusion in any meta-analysis.

### Data analysis

We conducted a narrative synthesis of the yield, reporting descriptive characteristics of included citations according to our meta-level variables, followed by synthesis of rates by continent, sex and study quality. Where there was sufficient data (*n* ≥ 5), we conducted random-effects meta-analysis (DerSimonian and Laird [19] method), since we anticipated high between-study heterogeneity [4]. Incidence rates were transformed onto the natural logarithmic scale alongside their corresponding standard errors (SEs). If no SE could be derived, we retained rates for narrative synthesis only. For assessments of differences in incidence by sex, we repeated this methodology for incidence rare ratios (IRRs) in men compared with women from available citations. Given the paucity of studies which reported standardized rates (i.e. for age and/or sex), we restricted meta-analyses to crude incidence comparisons only.

We tested heterogeneity using the *Q*-test and quantified this using the I^2^-statistic. In a change to the original protocol on PROSPERO, we did not analyse pooled estimates from meta-analyses because between-study heterogeneity was high (*I*^2^ ≥ 75%) [20]. We examined evidence of small study effects (including publication bias) by visual inspection of funnel plots (*N* ≥ 5) and formal testing using Egger’s test when sufficient estimates (*N* ≥ 10) were available.

Citations were managed in Mendeley (version 1.17.12) and extracted data were managed in an Excel spreadsheet. Meta-analyses were conducted in R using the ‘meta’ package [21] by one author (TD).

### Role of the funding source and ethical considerations

Funders had no role in any aspect of this study including the decision to submit the paper for publication. No ethics approval was required for this study, as no original data was gathered.

## Results

### Characteristics of included studies

**Fig. 1:**
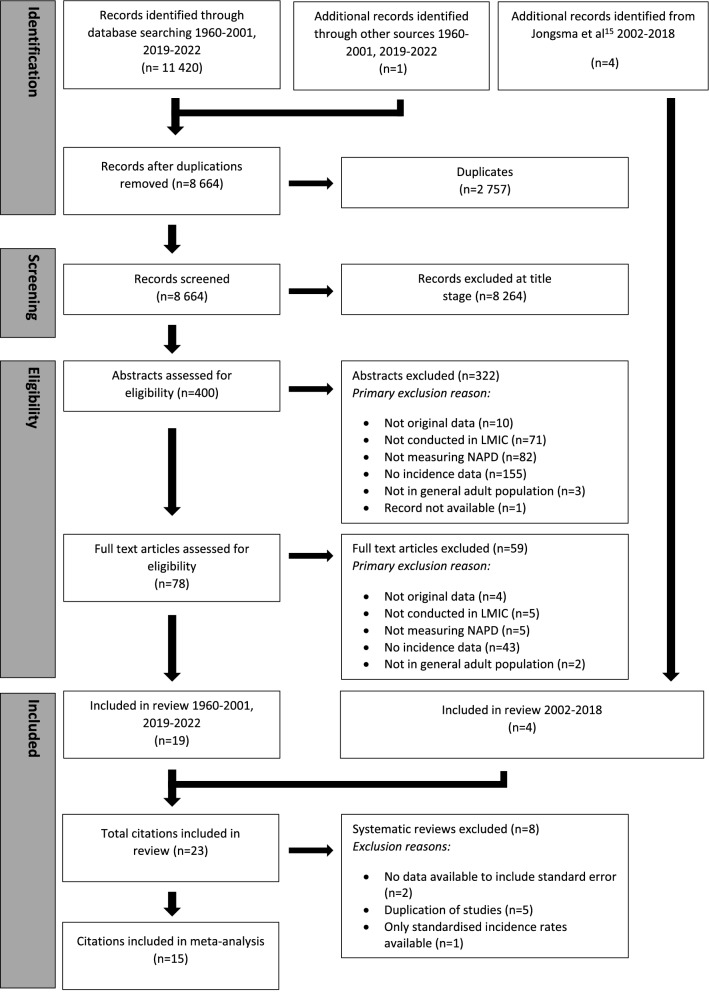
PRISMA flow diagram

We retrieved 8 664 records after removal of duplicates, of which 23 met inclusion criteria (Fig. 1) [7–10, 15, 22–39]. Four citations reported overlapping data from two centres (India, USSR) of the WHO “10-country study” [8, 9, 29, 30]; where relevant, we used data from Jablensky et al. [8] as our core citation (Supplemental Materials, Sect. 5). Two further citations [33, 34] provided overlapping incidence data from a study in Haidian District, Beijing, with Chen et al. [33] providing the core citation. Two studies provided overlapping incidence data from Ribeirão Preto (Brazil) [37] with Jongsma et al. [15] providing the core citation. Despite extensive searches, including contacting the authors of an earlier global systematic review of the incidence of schizophrenia [3], we were unable to obtain the full text of two core citations which met our inclusion criteria based on abstract information [31, 32]; we were able to include data from one of these [32] based on available data published in that review [3].

From 18 core citations [7, 8, 10, 15, 22–28, 31–33, 35, 36, 39], 15 (83.3%) had sufficient data available to include in meta-analyses [7, 8, 10, 15, 22–28, 33, 35, 39], with two studies not reporting sample sizes or standard errors alongside incidence rates [31, 32] and a further study providing standardised incidence rates only [36]. The median sample size from the remaining 15 studies was 320 cases (interquartile range: 70–809; range: 15 cases of schizophrenia in India [22] to 178 173 cases in Brazil [25]; Table 1). Core citations contributed original incidence data from 19 separate settings (Morgan et al. [39] and Jablensky et al. [8] each included data from two sites) in ten LMICs in South America (*N* = 8; 50.0%; Brazil [7, 15, 25, 36], Suriname [23, 27], Colombia [38] and Costa Rica [26]), Asia (*N* = 5; 35.7%; India [8, 22, 39], China [33, 35]), Africa (*N* = 3; 21.4%; Nigeria [28, 39], South Africa [24]), Europe (*N* = 3; 21.4%; Moscow, former USSR [8, 31, 32]), and North America (*N* = 1; 6.7%; Jamaica [10]). No studies were identified from LMICs in Australasia. Median year of case ascertainment was 1992 (IQR: 1979–2005), ranging from 1967 [25] to 2013 [15]. All core citations used a first contact or admission design, except for two household surveys in India [22] and China [33]. Only four citations (26.7%) reported age-sex [15, 23, 27, 39] (and in one study [15], age-sex-ethnicity) standardized rates.

**Table 1:** Characteristics of included studies on the incidence of non-affective psychotic disorders in low and middle-income countries, 1960–2019

Lead Author (year)	Country	Study Name^a^	Study Type	Diagnostic classification system	Diagnostic outcomes (codes)^b^	Mid-year recruitment period	Age range	Number of cases	Denominator	Incidence rate^f^	Study quality
Burns (2008) [24]	South Africa	–	First contact	DSM-IV	All psychotic disorders	2005	15–49	160	508,275	31.5^h^	4
Caetano (1981) [25]	Brazil	–	First contact	NR	Schizophrenia	1967	0–60+	178,173	688,803,925	26.0^h^	2.5
Chen (1984) [33]	China	–	Household survey	ICD-9, DSM-III	Schizophrenia	1978	NR	34	304,150	11.5^h^	3
Da Rocha (2021) [37]	Brazil	–	First admission	ICD-10	Non-affective disorders (F20-29)	2007	18+	1,549,298	NR	82,9^j^	
Del Ben (2019)[37]	Brazil	EU-GEI	First contact and leakage study	ICD—10	All psychotic disorders (295.xx, 297.1, 298.8, 296.04, 296.44, 296.64)	2013	18–64	588	3,071,862	19,14	6
Handal (1997) [26]	Costa Rica	–	First admission	ICD-9	Schizophrenia (295.0–295.9)	1980	15+	2,934	6,087,137	48.2^g^	3
Hanoeman (2002) [27]	Suriname	–	First admission	DSM-III-R	Non-affective (schizophrenia and schizophreniform) disorders	1992	15–54	73	453,384	16.0^g^	4
Hickling (1995) [10]	Jamaica	–	First contact	ICD-9/CATEGO	Non-affective disorders (S? P and O) Schizophrenia (S +)	1992	15–54	320 285	1,355,932 1,355,932	23.6^g^ 20.9^g^	3.5
Huang (1990) [36]	China	–	First admission	NR	Schizophrenia	1983	All	1359	5,461,950	24.9^h^	3
Ihezue (1982) [28]	Nigeria	–	First admission	NR	Schizophrenia	1980	All	67	480,183	14.0^h^	1
Jablensky (1992) [8]	India, USSR^c^	WHO-10	First contact and leakage study	ICD-9/CATEGO	Non-affective psychoses (ICD-9: 291.3, 291.5, 292.1, 295, 297, 298.3, 298.4, 298.9) Schizophrenia (CATEGO S +)	1979 (India) 1980 (USSR)	15–54	406 136	1,275,000 1,275,000	31.8^h^ 10.7^h^	6
Jongsma (2018) [15]	Brazil	EU-GEI	First contact and leakage study	ICD-10	All psychotic disorders (F20-33) Non-affective psychoses (20–29)	2013	18–64	565 389	2,631,689 2,631,689	21.5^g^ 14.8^g^	6
Liberman (1974) [32]	USSR^c^	IPSS	First contact	ICD-8/CATEGO	Schizophrenia	1968	NR	NR	NR	NR	-^e^
Menezes (2007) [7]	Brazil	–	First contact and leakage study	DSM-IV	All psychotic disorders (291.1, 295.1–295.9, 296.0, 296.24, 296.4, 298.8, 298.9) Non-affective psychoses (291.1, 295.1–295.9, 298.8, 298.9)	2003	18–64	367 231	2,315,203 2,315,203	15.9^g^ 10.0^g^	6
Morgan (2016) [39]	India, Nigeria	INTREPID-I	First contact and leakage	ICD-10	All psychotic disorders Schizophrenia (F20.X)	2013	18–64	112 80	284,201 284,201	39.4^h^ 28.2^h^	5
Rajkumar (1993) [22]	India	–	Household survey and leakage study^d^	ICD-9	Schizophrenia (295)	1988	15+	15	42,857	35.0^g^	6
Rotshteĭn (1982) [31]	USSR^c^	–	NR	NR	Paranoid Schizophrenia	NR	NR	NR	NR	NR	–^e^
Sartorius (1986) [9]	India, USSR^c^	WHO-10	First contact	ICD-9/CATEGO	Non-affective psychoses (ICD-9: 291.3, 291.5, 292.1, 295, 297, 298.3, 298.4, 298.9) Schizophrenia (CATEGO S +)	1979 (India) 1980 (USSR)	15–54	NR	NR	NR	6
Selten (2005) [23]	Suriname	–	First contact	DSM-IV	Non-affective disorders	2002	15–54	64	380,952	16.8^g^	4
Shen (1987) [34]	China	As Chen [33]	Household survey	NR	Schizophrenia	1977	NR	NR	NR	NR	2
Song (2022) [38]	Colombia	–	First contact	ICD-10	Schizophrenia	2012	18–90	1053	2,340,000^h^	45.0	2
Tsirkin (1987) [30]	USSR^c^	WHO-10	NR	NR	Schizophrenia	1980	NR	NR	NR	NR	–^e^
Wig (1993) [29]	India	WHO-10	First contact and leakage	ICD-9	Schizophrenia	1979	15–54	209	535,622	39.0^h^	6

*ICD* International Classification of Diseases, *DSM* diagnostic and statistical manual, *USSR* United Soviet Socialist Republic, *WHO-10* WHO 10-country study, *IPSS* International Pilot Study of Schizophrenia, *EUGEI* European Network of National Schizophrenia Networks Studying Gene-Environment Interactions study, *NR* not reported

^a^Colloquial name of larger study from which citation originates

^b^Where no diagnostic codes are displayed, none were reported

^c^The United Soviet Socialist Republic was dissolved in 1991. Catchment area for all USSR studies was Moscow, in present-day Russia

^d^The leakage study here included referrals by GPs, traditional medicine practitioners, faith healers as well as hospital records

^e^Study quality was only assessed for citations for which a full-text was available

^f^Incidence per 100,000 person-years

^g^Reported incidence rate

^h^Derived

^j^Standardised for age and sex

### Incidence of psychotic disorders

**Fig. 2:**
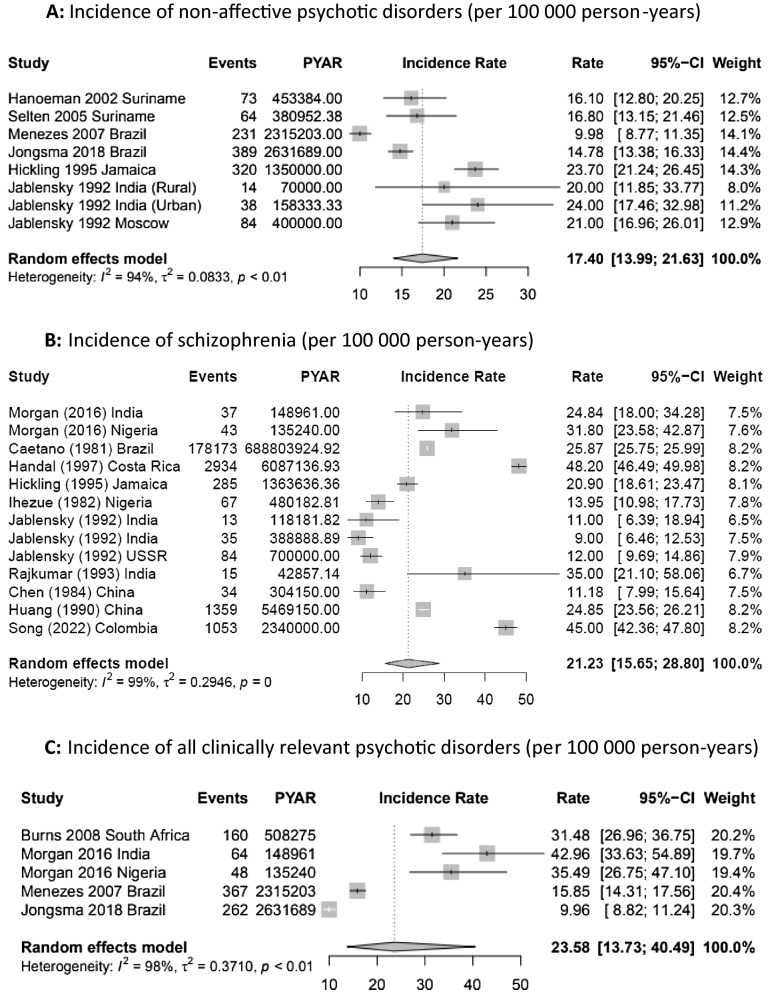
Forest plots of incidence of selected psychotic disorders in LMICs

Six core citations (40.0%) included eight separate estimates for NAPD in five LMICs (Fig. 2a) [7, 8, 10, 15, 23, 27]. We observed a fourfold variation in crude incidence, from 10.0 cases per 100,000 person-years in Sao Paulo, Brazil (95%CI 8.7–11.4) [7] to 42.0 (95%CI 32.2–54.8) in rural Chandigarh in India [8]. Heterogeneity was high (*I*^2^: 97.3%, *Q*: 259.6; *p* < 0.01).

Schizophrenia was the most frequently studied diagnostic outcome with 13 unique rates reported from ten (55.6%) core citations from eight LMICs (Fig. 2b) [8, 10, 22, 25, 26, 28, 32, 33, 39]. Crude incidence varied approximately fivefold, from 9.0 cases per 100,000 person-years (95%CI 6.5–12.5) in India [8] to in 48.2 (95%CI 46.5–50.0) in Costa Rica [25]. Heterogeneity was high (*I*^2^: 99.3%; *Q*: 1600.8, *p* < 0.0001).

Four core citations (26.7%) reported the incidence of all clinically relevant psychotic disorders in Brazil, India, Nigeria and South Africa (Fig. 2c) [15, 23, 24, 39]. Rates varied from 15.8 per 100,000 person-years (95%CI 14.3–17.6) in Sao Paulo, Brazil, to 45.9 (95%CI 34.5–57.3) in Chengalpattu, India [39]. Heterogeneity was high (*I*^2^: 98.0%; *Q*: 99.9, *p* < 0.01).

Four core citations (26.7%) also reported incidence data for the affective psychoses from four settings [7, 10, 15, 25], three in Brazil [7, 15, 25] and one in Jamaica [10]. Rates of affective psychosis in Brazil were similar in three independent samples and time points, ranging from 5.9 per 100,000 person-years (95%CI: 5.7–7.7) in a population-based study in Sao Paulo [7] to 7.3 (95%CI 7.2–7.4) in a nationwide study based on hospitalised admissions [25]. In Jamaica, Hickling and Rodgers-Johnson [10] reported a lower incidence of affective psychoses, derived from 15 cases with a crude rate of 1.1 per 100,000 person-years (95%CI 0.7–1.8).

### Variance by continent

#### The Americas

Nine core citations estimated incidence rates in LMIC settings in the Americas, including four from Brazil (two national [25, 36], two catchment area-based [7, 15]), two national studies from Suriname [23, 27], two national studies in Costa Rica [26] and Jamaica [10], and a study in the state of Caldas, Colombia [38]. In Brazil, a nationwide study of all first psychiatric admissions between 1960 and 1974 reported the treated incidence of schizophrenia and affective psychoses over time for men and women separately [25]. Reported crude treated rates of both sets of disorders generally increased over the time period, with overall rates of 25.9 cases of schizophrenia per 100,000 person-years (95%CI 25.7–26.0), and 7.3 cases of affective psychoses (95%CI 7.2–7.4). Incidence rates of schizophrenia were higher in this study than the overall rate of all clinically relevant psychotic disorders estimated from two catchment area studies in Sao Paulo (15.8 per 100,000 person-years; 95%CI 14.3–17.6) [7], and Ribeirao Preto (21.5; 95%CI 19.8–23.3) [15], which—in contrast to the nationwide study [25]—both employed standardised research-based diagnoses, population-based case finding approaches, a leakage design to ascertain potentially missed cases by the initial screen, and restricted the age range to 18–64 years old. A further nationwide study detailing psychiatric hospitalisations reported an age-sex standardised incidence rate of NAPD of 82.9 (95%CI 71.6–94.1) per 100,000 person-years, based on an assessment of clinical records only [36].

Two separate nationwide studies of the incidence of NAPDs in Suriname reported similar crude (16.1 [27] and 16.8 [23] per 100,000 person-years, respectively) and age-sex standardised rates (16.8 [27] and 17.7 [23], respectively) for people aged 15–54 years old, albeit using different designs. Hanoeman et al.’s earlier study [27] was based on all first admissions to the country’s only psychiatric hospital, whilst Selten et al.’s [23] study also identified cases through primary care. Hickling et al. [10] reported higher incidence rates of NAPD (23.6 per 100,000 person-years; 95%CI: 21.2–26.3) and schizophrenia (20.9; 95%CI 18.6–23.5) in a comparable nationwide study in Jamaica. An earlier nationwide study in Costa Rica reported high hospitalised rates of schizophrenia for people aged 15 years and older (48.2 per 100,000 person-years; 95%CI 46.5–50.0) based on administrative data using clinical diagnoses, with a similar estimate of crude incidence (45 per 100,000 person-years; 95%CI 42.4–47.8) found using a similar design between 2005 and 2018 in Colombia [38].

#### Asia

Five core citations estimated the incidence of NAPD in Asia, three based in India [8, 22, 39], and two in China [33, 35]. Data from the WHO 10-country study [8] (also [9, 29]) suggested that crude incidence rates of NAPD were similar in rural and urban Chandigarh (rural: 42.0 per 100,000 person-years; 95%CI 32.2–54.8; urban: 35.0; 95%CI 29.9–41.0), and perhaps slightly higher than in Moscow (former USSR; 28.0; 95%CI 24.4–32.2). These patterns were similar for the incidence of schizophrenia. Comparably high crude and age-sex standardised incidence rates of all psychotic disorders (crude [derived]: 43.0; 95%CI 33.6–54.9; standardised: 45.9; 95%CI 34.5–57.3) and schizophrenia (crude [derived]: 24.8: 18.0–34.3; standardised: 27.2; 95%CI 18.3–36.0) were reported in provisional data from the INTREPID-I study in Chengalpattu, India [39]. In China, one study from Haidian District (Beijing) reported a lower treated incidence of schizophrenia (11.2; 95%CI 8.0–15.6), ascertained between 1975 and 1981 (also see [34]), while a second study from Guangdong province reported a crude incidence of 24.9 (95%CI 23.6–26.1) with cases recruited between 1978 and 1987 [35].

#### Africa

Three core citations provided incidence data in Africa: two from Nigeria [28, 39], and a third in South Africa [24]. In Nigeria, a 30-day study conducted in April 1980 reported all treated cases diagnosed with schizophrenia for the first time in the only psychiatric hospital in the state of Anambra [28]. We estimated the derived incidence as 14.0 new cases per 100,000 person-years (95%CI 11.0–17.7; see Supplemental Materials, Sect. 7). A more recent study [39] in Ibadan, Nigeria, used a population-based case finding approach to estimate the crude (derived) and age-sex standardised incidence of schizophrenia as 31.8 per 100,000 person-years (95%CI 23.6–42.9) and 27.5 (95%CI 19.1–36.0), respectively. Corresponding rates for all clinically relevant psychotic disorders were 35.5 (95%CI 26.7–47.1) and 31.2 (95%CI 22.2–40.3), respectively [39]. This was similar to the crude treated incidence reported in the District of uMgungundlovu, KwaZulu-Natal, South Africa (31.5; 95%CI 27.0–36.8) [24].

#### Europe

We identified five citations which provided estimates of incidence rates in the former USSR (all Moscow [8, 9, 30–32]), including three core citations [8, 31, 32]. The earliest report estimated the overall treated incidence of ICD-8 schizophrenia in people aged 15–44 years as 19.1 per 100,000 person-years (no SE provided) [32], as one of the field centres participating in the WHO International Pilot Study of Schizophrenia. A lower incidence in the population aged 15–54 was reported in the WHO 10-country study as 12.0 (95%CI 9.7–14.9) [8], with a higher rate of NAPDs in that sample (28.0; 95%CI 24.4–32.2). Finally, in 1982, Rotshtein [31] reported the incidence of paranoid schizophrenia as 1.7 per 100,000 person-years (no SE provided) [31].

### Variance by sex

Five core citations reported incidence rates for men and women separately [8, 10, 15, 25, 32]. Point estimates of incidence for all psychotic disorders [15], and schizophrenia [10, 25, 32] were generally higher for men than women, except in the WHO 10-country study [8], which found no consistent evidence of this effect [8] (Supplemental Materials, Sect. 8). In Brazilian national data, there was evidence of higher rates of affective psychoses in women than men across a 14-year period [25].

### Quality appraisal

Study quality ranged from one to six (out of seven; Table 1, Supplemental Table 2), with a median of four (IQR: 3–6) from 16 of the 18 core citations for which a full-text review could be performed (Supplemental Materials, Sect. 6). We found no evidence of correlation (*ρ* = 0.32; *p* = 0.23) between reported study quality and median year of case ascertainment (Supplemental Fig. 1). We found no evidence of substantive correlation between incidence rates of schizophrenia and study quality (*ρ* = − 0.29; *p* = 0.34) or time period (*ρ* = 0.41; *p* = 0.16). We could not perform formal meta-regression on these data due to the small number of available data points.

### Small study effects

Funnel plots for NAPD, schizophrenia and all FEP (Supplemental Figs. 2–4) did not provide evidence of small study effects for patterns of incidence on these outcomes. We only had sufficient citations to formally test small study effects via an Egger’s test for schizophrenia (*N* = 13; *p* = 0.70), which suggested no evidence of funnel plot asymmetry. There may, however, have been some evidence that smaller studies of lower rates of all clinically relevant psychotic disorders were absent from the published literature (Supplemental Fig. 4).

## Discussion

### Summary of main results

Our systematic review identified 23 citations published over a 60-year period on the incidence of NAPD in general adult population studies conducted in LMICs. This yield was limited to 19 settings from just ten countries, highlighting the dearth of evidence on the incidence and distribution of NAPD in LMICs. This paucity of evidence was further compounded by a lack of nationwide samples (only five settings) limiting knowledge to a small number of regions. Three-quarters of the identified studies were based on data collected with a mid-point of case ascertainment before 2003, and 50% before 1990. Studies were also characterised by high heterogeneity in research methods, diagnostic procedures and outcomes, and study quality.

### Strengths and limitations of our review

Strengths of our review included an inclusive search strategy, based on previously-validated methods [2, 4] which followed the principles of the Cochrane Library, PRISMA guidelines, and prospective registration of our protocol on PROSPERO (Supplemental Materials, Sect. 9).

Limitations of our review include omission of data from one citation which may have met our inclusion based on its title (no abstract available) [40], despite exhaustive searches. Whilst we specified no exclusion criteria based on language, we limited our search to English language databases, and cannot exclude omission of relevant studies not indexed by these repositories. Despite this, we found no strong evidence of small study effects. We grouped studies based on broad diagnostic criteria, which masked variability in exact outcomes studied and diagnostic classification systems used in individual citations (Table 1). Although our primary diagnostic outcome was NAPD, we do not believe we will have omitted many studies of other clinically relevant non-organic psychotic disorders, since our search terms included “schizophrenia”, “psychosis”, “mental illness/disorder” and related variants (Supplemental Materials, Sect. 1). The study quality tool we used may have not captured total study quality; for example, it did not allow us to rate citations which failed to report the numerator (case) sample sizes for reported rates [8–10, 29–32]. The Cochrane filter on LMICs is based on current categorisations, making it possible we omitted studies conducted in countries which were subsequently redefined as HICs. Finally, we might have missed studies that do not explicitly state incidence rates, but which might contain sufficient information to derive them. However, at each stage of the selection process we aimed to be inclusive and only excluded true negatives. At full-text stage this included ascertaining if citations included sufficient data to derive incidence rates.

### Meaning of the findings in context

To the best of our knowledge, this is the first standalone systematic review of the incidence of psychotic disorders in LMICs. Previous international reviews have not separately synthesised results from LMIC settings [3, 4], and either covered a narrow time period [4] or were restricted to schizophrenia [3]. The four- to fivefold variation in rates of NAPD and schizophrenia observed in LMICs is consistent with variance reported in earlier global reviews [3, 4], and with a synthesis of the available evidence in a multi-country study [29].

We observed heterogeneous methods and incidence estimates in LMICs, similarly to Morgan et al. [39], though with a more up-to-date summary of the available evidence. There was limited evidence that study quality had risen over time, although study quality was not correlated with variation in reported rates. Nevertheless, the comparison of incidence rates of psychotic disorders is predicated on sufficient commonality across methods to permit valid inferences about the distribution of psychotic disorders by person and place. While some basic tenets were evident across most studies identified here (defined catchment areas, accurate denominator, reporting of inclusion criteria), other methodological features were more variably applied (Supplemental Table 2).

Some of these issues may have particularly profound effects on estimating and comparing incidence rates in LMICs. For example, studies reliant on detecting cases solely presenting to psychiatric care [10, 25, 26, 28, 33] may underestimate the true population incidence of psychotic disorders, particularly in countries where formal mental health resources are more limited, or where help-seeking takes place in traditional or informal settings. In our review, evidence on this issue was limited. Two nationwide studies in Suriname reported similar rates of NAPD a decade apart [23, 27], whether case finding was restricted to secondary psychiatric care or extended to include primary care [23]. In contrast, two studies in Nigeria estimated a twofold difference in schizophrenia incidence depending on whether case ascertainment was restricted to first contact with psychiatric care [28] or used a population-based case finding approach [39]. This [39, 41], and other incidence initiatives underway in South Africa [42] and Iran [43], suggest that epidemiological methods can be adapted in different contexts to enable reliable case ascertainment to accurately estimate incidence in different contexts.

Methodological heterogeneity in incidence studies of psychotic disorders is by no means limited to LMICs [4], but has arguably become an endemic issue [15, 44], inhibiting progress in the field. Even where studies adopt common methodological procedures, our review highlights how their variable operationalisation may hinder meaningful comparisons. For example, although most studies defined their age ranges, this was variably applied (i.e. 15–44 [32], 15–49 [24], 15–54 [8–10, 23, 27, 29], 18–64 [7, 15, 39], 18–90 [38], the entire adult population [22, 26, 33], or entire population [25]). Finally, comparisons in this review were largely limited to crude rates, with only four core citations [15, 23, 27, 39] (26.7%) reporting age-sex standardised rates, an issue not limited to LMICs. Universal reporting of standardised rates will facilitate better quantification and exploration of heterogeneity in incidence across different settings.

To make meaningful progress in this field of psychosis epidemiology, we recommend two priorities. First, more investment is made to estimate the true incidence of all non-organic psychotic disorders worldwide, expanding beyond the relatively narrow set of countries where research has been conducted to date. Whereas around 87% of the global population lives in LMICs, only 2.4% of mental health research funding is currently spent on examining mental health in these settings [45]. Second, we recommend the development and adoption of a common epidemiological framework and guidelines for epidemiological studies to estimate the incidence of psychotic disorders reliably and comparably across diverse contexts. These priorities should be aligned, by funding ambitious, global consortia to establish a network of researchers collaborating to simultaneously estimate the incidence of psychosis in different settings, providing a foundation for further investigation of the aetiological determinants of, and inequalities in, variability in risk, course, and outcomes following the onset of psychosis across the globe. These efforts would aid and inform other global initiatives, including the World Health Survey and Global Burden of Diseases, which respectively, provide new understanding about the prevalence of subclinical psychotic symptoms [12, 13] and model-based projections of the likely incidence of disorder in different countries [46]. All such approaches are imperfect, but triangulating their findings would overcome limitations of current epistemological inferences about the distribution and determinants of the incidence of psychotic disorders, predominantly based on evidence generated in HICs.

## Supplementary Information

Below is the link to the electronic supplementary material.

Supplementary file1 (DOCX 386 KB)

## Data Availability

The dataset generated and analysed in this study is publicly available via the Open Science Framework.Link to the project page: https://osf.io/ahb3q/
