# Supplementary material for: The incidence of non-affective psychotic disorders in low and middle-income countries: a systematic review and meta-analysis
Source: Soc Psychiatry Psychiatr Epidemiol. 2022 Dec 22;58(4):523–36. doi: 10.1007/s00127-022-02397-6 (PMC10066134; doi:10.1007/s00127-022-02397-6)
Supplement: Supplementary file 1 — Supplementary file1 (DOCX 386 KB) [file 127_2022_2397_MOESM1_ESM.docx]

Supplemental Material to John-Baptiste Bastien R, Ding T, Gonzalez-Valderamma A, Valmaggia L, Kirkbride JB and Jongsma HE (2021) The incidence of non-affective disorders in Low and Middle Income Countries: a systematic review and meta-analysis.

1. **Search terms**

Searches were initially run from database conception to 14 April 2020, as detailed in the paper methodology. Following peer review, searches were re-run with limits applied from 15April 2020 to 31 May 2022. Numbers in brackets below indicate yields from initial search. Search yields for the later search are not shown below, but can be found in our Open Science Framework repository at [10.17605/OSF.IO/AHB3Q](https://doi.org/10.17605/OSF.IO/AHB3Q). Our search terms were developed in consultation with an academic librarian, and adapted and applied to the following databases:

- 1. **Embase Search strategy**

Database: Embase Classic+Embase <1947 to 2020 April 14>

Search Strategy:

--------------------------------------------------------------------------------

1 schizo*.tw. (192067)

2 psychotic.tw. (54467)

3 psychos?s.tw. (68742)

4 ((severe or serious or chronic) and mental and (illness* or disorder*)).tw. (45535)

5 SMI.tw. (6692)

6 chronic psychosis.tw. (474)

7 schizophrenia/ or schizophrenia spectrum disorder/ or catatonic schizophrenia/ or paranoid schizophrenia/ or residual schizophrenia/ (189888)

8 cannabis-induced psychosis/ or drug induced psychosis/ or experimental psychosis/ or alcohol psychosis/ or paranoid psychosis/ or psychosis/ or childhood psychosis/ or acute psychosis/ or methamphetamine-induced psychosis/ or cocaine-induced psychosis/ (106130)

9 psychosis/ or brief psychotic disorder/ or endogenous psychosis/ or experimental psychosis/ (101317)

10 delusion* disorder.tw. (1191)

11 1 or 2 or 3 or 4 or 5 or 6 or 7 or 8 or 9 or 10 (360730)

12 (inciden* or epidemiolog*).tw. (1706277)

13 ((first* or 1st) adj3 episode*).tw. (28767)

14 ((first* or 1st*) adj3 hospital* adj3 (contact* or admission* or admit*)).tw. (2346)

15 (case and register*).tw. (26780)

16 (prospective* or population* or communit* or survey*).tw. (4318776)

17 Developing Country.sh. (95429)

18 (Africa or Asia or Caribbean or West Indies or South America or Latin America or Central America).hw,ti,ab,cp. (355019)

19 (Afghanistan or Albania or Algeria or Angola or Argentina or Armenia or Armenian or Azerbaijan or Bangladesh or Benin or Byelarus or Byelorussian or Belarus or Belorussian or Belorussia or Belize or Bhutan or Bolivia or Bosnia or Herzegovina or Hercegovina or Botswana or Brasil or Brazil or Bulgaria or Burkina Faso or Burkina Fasso or Upper Volta or Burundi or Urundi or Cambodia or Khmer Republic or Kampuchea or Cameroon or Cameroons or Cameron or Camerons or Cape Verde or Central African Republic or Chad or Chile or China or Colombia or Comoros or Comoro Islands or Comores or Mayotte or Congo or Zaire or Costa Rica or Cote d'Ivoire or Ivory Coast or Cuba or Djibouti or French Somaliland or Dominica or Dominican Republic or East Timor or East Timur or Timor Leste or Ecuador or Egypt or United Arab Republic or El Salvador or Eritrea or Ethiopia or Fiji or Gabon or Gabonese Republic or Gambia or Gaza or Georgia Republic or Georgian Republic or Ghana or Gold Coast or Grenada or Guatemala or Guinea or Guiana or Guyana or Haiti or Honduras or India or Maldives or Indonesia or Iran or Iraq or Jamaica or Jordan or Kazakhstan or Kazakh or Kenya or Kiribati or Kosovo or Kyrgyzstan or Kirghizia or Kyrgyz Republic or Kirghiz or Kirgizstan or Lao PDR or Laos or Lebanon or Lesotho or Basutoland or Liberia or Macedonia or Madagascar or Malagasy Republic or Malaysia or Malaya or Malay or Sabah or Sarawak or Malawi or Nyasaland or Mali or Marshall Islands or Mauritania or Mauritius or Agalega Islands or Mexico or Micronesia or Middle East or Moldova or Moldovia or Moldovian or Mongolia or Montenegro or Morocco or Ifni or Mozambique or Myanmar or Myanma or Burma or Namibia or Nepal or Netherlands Antilles or Nicaragua or Niger or Nigeria or Muscat or Pakistan or Palestine or Paraguay or Peru or Philippines or Philipines or Phillipines or Phillippines or Romania or Rumania or Roumania or Russia or Russian or Rwanda or Ruanda or Saint Lucia or St Lucia or Saint Vincent or St Vincent or Grenadines or Samoa or Samoan Islands or Navigator Island or Navigator Islands or Sao Tome or Senegal or Serbia or Montenegro or Sierra Leone or Slovenia or Sri Lanka or Ceylon or Solomon Islands or Somalia or South Africa or Sudan or Suriname or Surinam or Swaziland or Syria or Tajikistan or Tadzhikistan or Tadjikistan or Tadzhik or Tanzania or Thailand or Togo or Togolese Republic or Tonga or Tunisia or Turkey or Turkmenistan or Turkmen or Uganda or Ukraine or USSR or Soviet Union or Union of Soviet Socialist Republics or Uzbekistan or Uzbek or Vanuatu or New Hebrides or Venezuela or Vietnam or Viet Nam or West Bank or Yemen or Yugoslavia or Zambia or Zimbabwe or Rhodesia).hw,ti,ab,cp. (3491845)

20 ((developing or less* developed or under developed or underdeveloped or middle income or low* income or underserved or under served or deprived or poor*) adj (countr* or nation? or population? or world)).ti,ab. (125544)

21 ((developing or less* developed or under developed or underdeveloped or middle income or low* income) adj (economy or economies)).ti,ab. (706)

22 (low* adj (gdp or gnp or gross domestic or gross national)).ti,ab. (346)

23 (low adj3 middle adj3 countr*).ti,ab. (18249)

24 (lmic or lmics or third world or lami countr*).ti,ab. (8985)

25 transitional countr*.ti,ab. (228)

26 or/17-25 (3725827)

27 12 or 13 or 14 or 15 or 16 (5498283)

28 11 and 26 and 27 (9569)

29 limit 28 to yr="1960 - 2002" (1429)

30 limit 28 to yr="2017 - 2019" (1994)

31 29 or 30 (3423)

- 1. **Medline search strategy**

Database: Ovid MEDLINE(R) and Epub Ahead of Print, In-Process & Other Non-Indexed Citations and Daily <1946 to April 14, 2020>

Search Strategy:

--------------------------------------------------------------------------------

1 schizo*.tw. (138691)

2 psychotic.tw. (32812)

3 psychos?s.tw. (43003)

4 ((severe or serious or chronic) and mental and (illness* or disorder*)).tw. (30125)

5 SMI.tw. (4473)

6 Schizophrenia/ or Schizophrenia, Catatonic/ or Schizophrenia, Childhood/ (101226)

7 exp Psychotic Disorders/ (51568)

8 Psychoses, Substance-Induced/ (5263)

9 delusion* disorder.tw. (693)

10 1 or 2 or 3 or 4 or 5 or 6 or 7 or 8 or 9 (241046)

11 (inciden* or epidemiolog*).tw. (1174489)

12 ((first* or 1st) adj3 episode*).tw. (17091)

13 ((first* or 1st*) adj3 hospital* adj3 (contact* or admission* or admit*)).tw. (1446)

14 (case and register*).tw. (16727)

15 (prospective* or population* or communit* or survey*).tw. (3128282)

16 11 or 12 or 13 or 14 or 15 (3950067)

17 Developing Countries.sh,kf. (85460)

18 (Africa or Asia or Caribbean or West Indies or South America or Latin America or Central America).hw,kf,ti,ab,cp. (274650)

19 (Afghanistan or Albania or Algeria or Angola or Argentina or Armenia or Armenian or Azerbaijan or Bangladesh or Benin or Byelarus or Byelorussian or Belarus or Belorussian or Belorussia or Belize or Bhutan or Bolivia or Bosnia or Herzegovina or Hercegovina or Botswana or Brasil or Brazil or Bulgaria or Burkina Faso or Burkina Fasso or Upper Volta or Burundi or Urundi or Cambodia or Khmer Republic or Kampuchea or Cameroon or Cameroons or Cameron or Camerons or Cape Verde or Central African Republic or Chad or Chile or China or Colombia or Comoros or Comoro Islands or Comores or Mayotte or Congo or Zaire or Costa Rica or Cote d'Ivoire or Ivory Coast or Cuba or Djibouti or French Somaliland or Dominica or Dominican Republic or East Timor or East Timur or Timor Leste or Ecuador or Egypt or United Arab Republic or El Salvador or Eritrea or Ethiopia or Fiji or Gabon or Gabonese Republic or Gambia or Gaza or Georgia Republic or Georgian Republic or Ghana or Gold Coast or Grenada or Guatemala or Guinea or Guiana or Guyana or Haiti or Honduras or India or Maldives or Indonesia or Iran or Iraq or Jamaica or Jordan or Kazakhstan or Kazakh or Kenya or Kiribati or Kosovo or Kyrgyzstan or Kirghizia or Kyrgyz Republic or Kirghiz or Kirgizstan or Lao PDR or Laos or Lebanon or Lesotho or Basutoland or Liberia or Macedonia or Madagascar or Malagasy Republic or Malaysia or Malaya or Malay or Sabah or Sarawak or Malawi or Nyasaland or Mali or Marshall Islands or Mauritania or Mauritius or Agalega Islands or Mexico or Micronesia or Middle East or Moldova or Moldovia or Moldovian or Mongolia or Montenegro or Morocco or Ifni or Mozambique or Myanmar or Myanma or Burma or Namibia or Nepal or Netherlands Antilles or Nicaragua or Niger or Nigeria or Muscat or Pakistan or Palestine or Paraguay or Peru or Philippines or Philipines or Phillipines or Phillippines or Romania or Rumania or Roumania or Russia or Russian or Rwanda or Ruanda or Saint Lucia or St Lucia or Saint Vincent or St Vincent or Grenadines or Samoa or Samoan Islands or Navigator Island or Navigator Islands or Sao Tome or Senegal or Serbia or Montenegro or Sierra Leone or Slovenia or Sri Lanka or Ceylon or Solomon Islands or Somalia or South Africa or Sudan or Suriname or Surinam or Swaziland or Syria or Tajikistan or Tadzhikistan or Tadjikistan or Tadzhik or Tanzania or Thailand or Togo or Togolese Republic or Tonga or Tunisia or Turkey or Turkmenistan or Turkmen or Uganda or Ukraine or USSR or Soviet Union or Union of Soviet Socialist Republics or Uzbekistan or Uzbek or Vanuatu or New Hebrides or Venezuela or Vietnam or Viet Nam or West Bank or Yemen or Yugoslavia or Zambia or Zimbabwe or Rhodesia).hw,kf,ti,ab,cp. (2845946)

20 ((developing or less* developed or under developed or underdeveloped or middle income or low* income or underserved or under served or deprived or poor*) adj (countr* or nation? or population? or world)).ti,ab. (98370)

21 ((developing or less* developed or under developed or underdeveloped or middle income or low* income) adj (economy or economies)).ti,ab. (536)

22 (low* adj (gdp or gnp or gross domestic or gross national)).ti,ab. (239)

23 (low adj3 middle adj3 countr*).ti,ab. (15784)

24 (lmic or lmics or third world or lami countr*).ti,ab. (7371)

25 transitional countr*.ti,ab. (159)

26 17 or 18 or 19 or 20 or 21 or 22 or 23 or 24 or 25 (3007630)

27 10 and 16 and 26 (6428)

28 limit 27 to yr="1960 - 2002" (1190)

29 limit 27 to yr="2017 - 2019" (1408)

30 28 or 29 (2598)

***************************

- 1. **PsycInfo search strategy**

Database: APA PsycInfo <1806 to April Week 1 2020>

Search Strategy:

--------------------------------------------------------------------------------

1 schizo*.tw. (129730)

2 psychotic.tw. (42329)

3 psychos?s.tw. (54071)

4 ((severe or serious or chronic) and mental and (illness* or disorder*)).tw. (33125)

5 SMI.tw. (2108)

6 exp Catatonic Schizophrenia/ or exp Childhood Schizophrenia/ or exp Acute Schizophrenia/ or exp Schizophrenia/ or exp Paranoid Schizophrenia/ (90061)

7 exp Psychosis/ or exp Acute Psychosis/ or exp Chronic Psychosis/ or exp Alcoholic Psychosis/ or exp Childhood Psychosis/ (115432)

8 delusion* disorder.tw. (856)

9 1 or 2 or 3 or 4 or 5 or 6 or 7 or 8 (204897)

10 (inciden* or epidemiolog*).tw. (128450)

11 ((first* or 1st) adj3 episode*).tw. (9201)

12 ((first* or 1st*) adj3 hospital* adj3 (contact* or admission* or admit*)).tw. (390)

13 (case and register*).tw. (2623)

14 (prospective* or population* or communit* or survey*).tw. (858745)

15 10 or 11 or 12 or 13 or 14 (945692)

16 Developing Country.sh. (0)

17 (Africa or Asia or Caribbean or West Indies or South America or Latin America or Central America).hw,ti,ab,cp. (36725)

18 (Afghanistan or Albania or Algeria or Angola or Argentina or Armenia or Armenian or Azerbaijan or Bangladesh or Benin or Byelarus or Byelorussian or Belarus or Belorussian or Belorussia or Belize or Bhutan or Bolivia or Bosnia or Herzegovina or Hercegovina or Botswana or Brasil or Brazil or Bulgaria or Burkina Faso or Burkina Fasso or Upper Volta or Burundi or Urundi or Cambodia or Khmer Republic or Kampuchea or Cameroon or Cameroons or Cameron or Camerons or Cape Verde or Central African Republic or Chad or Chile or China or Colombia or Comoros or Comoro Islands or Comores or Mayotte or Congo or Zaire or Costa Rica or Cote d'Ivoire or Ivory Coast or Cuba or Djibouti or French Somaliland or Dominica or Dominican Republic or East Timor or East Timur or Timor Leste or Ecuador or Egypt or United Arab Republic or El Salvador or Eritrea or Ethiopia or Fiji or Gabon or Gabonese Republic or Gambia or Gaza or Georgia Republic or Georgian Republic or Ghana or Gold Coast or Grenada or Guatemala or Guinea or Guiana or Guyana or Haiti or Honduras or India or Maldives or Indonesia or Iran or Iraq or Jamaica or Jordan or Kazakhstan or Kazakh or Kenya or Kiribati or Kosovo or Kyrgyzstan or Kirghizia or Kyrgyz Republic or Kirghiz or Kirgizstan or Lao PDR or Laos or Lebanon or Lesotho or Basutoland or Liberia or Macedonia or Madagascar or Malagasy Republic or Malaysia or Malaya or Malay or Sabah or Sarawak or Malawi or Nyasaland or Mali or Marshall Islands or Mauritania or Mauritius or Agalega Islands or Mexico or Micronesia or Middle East or Moldova or Moldovia or Moldovian or Mongolia or Montenegro or Morocco or Ifni or Mozambique or Myanmar or Myanma or Burma or Namibia or Nepal or Netherlands Antilles or Nicaragua or Niger or Nigeria or Muscat or Pakistan or Palestine or Paraguay or Peru or Philippines or Philipines or Phillipines or Phillippines or Romania or Rumania or Roumania or Russia or Russian or Rwanda or Ruanda or Saint Lucia or St Lucia or Saint Vincent or St Vincent or Grenadines or Samoa or Samoan Islands or Navigator Island or Navigator Islands or Sao Tome or Senegal or Serbia or Montenegro or Sierra Leone or Slovenia or Sri Lanka or Ceylon or Solomon Islands or Somalia or South Africa or Sudan or Suriname or Surinam or Swaziland or Syria or Tajikistan or Tadzhikistan or Tadjikistan or Tadzhik or Tanzania or Thailand or Togo or Togolese Republic or Tonga or Tunisia or Turkey or Turkmenistan or Turkmen or Uganda or Ukraine or USSR or Soviet Union or Union of Soviet Socialist Republics or Uzbekistan or Uzbek or Vanuatu or New Hebrides or Venezuela or Vietnam or Viet Nam or West Bank or Yemen or Yugoslavia or Zambia or Zimbabwe or Rhodesia).hw,ti,ab,cp. (185186)

19 ((developing or less* developed or under developed or underdeveloped or middle income or low* income or underserved or under served or deprived or poor*) adj (countr* or nation? or population? or world)).ti,ab. (17471)

20 ((developing or less* developed or under developed or underdeveloped or middle income or low* income) adj (economy or economies)).ti,ab. (371)

21 (low* adj (gdp or gnp or gross domestic or gross national)).ti,ab. (45)

22 (low adj3 middle adj3 countr*).ti,ab. (3068)

23 (lmic or lmics or third world or lami countr*).ti,ab. (1806)

24 transitional countr*.ti,ab. (64)

25 16 or 17 or 18 or 19 or 20 or 21 or 22 or 23 or 24 (211207)

26 9 and 15 and 25 (3479)

27 limit 26 to yr="1960 - 2002" (718)

28 limit 26 to yr="2017 - 2019" (631)

29 27 or 28 (1349)

***************************

- 1. **Web of Science search strategy**

A comparable search strategy was developed to extract citations from Web of Science. Due to the way in which searches are implemented in Web of Science, this relied on running multiple combinations of the aforementioned search terms manually, before compiling a final set of citations (see our online, open access database for initial yield of citations). Due to this complexity, the exact search history used is not included here, but mirrored the searches performed above.

1. **Citation Screening**

Given the large number of citations retrieved by our search terms (Supplemental Materials, Section 1), three authors (RJ-BB, JBK, HEJ) screened a third of the retrieved titles each to assess eligibility. Citations which could not be positively excluded based on their titles were forwarded for abstract review, independently performed by RJ-BB and HEJ, with discrepancies resolved by JBK. Definite or possible citations were forwarded for full text review, conducted independently by RJ-BB and HEJ with discrepancies resolved by JBK, to identify a final set of citations which met our inclusion criteria. Screening of citations published between April 2020 – May 2022 was conducted by a sole reviewer (JBK), following peer review.

1. **Diagnostic classification systems**

The diagnostic classification systems used in the identified citations for this review varied from the International Classification of Diseases [ICD], ninth and tenth revisions, to the Diagnostic and Statistical Manual [DSM], versions III-R and IV. Where no diagnostic classification was reported, we relied on the nomenclature used to define the outcomes under study reported in each citation. Consistent with our earlier reviews,^2,4^ we assumed sufficiently commonality across systems to permit comparison of rates of the same diagnostic outcomes.

1. **Study quality scoring methodology**

We assessed study quality according to seven quality criteria for incidence studies we have previously designed and published for systematic reviews of the incidence of psychotic disorders.^2,4^ These include (i) reporting of a defined catchment area; (ii) accurate reporting and reliable source of denominator data; (iii) evidence of a population-based case-finding approach; (iv) standardised research diagnosis used; (v) masking (of the clinician) to demographic variables such as the ethnicity of the participant; (vi) inclusion criteria clearly stated, and; (vii) inclusion of a leakage study to identify potentially missed cases during the initial case ascertainment phase.

1. **Jablensky et al^8^ – The WHO “10-Country study”**

Although this landmark World Health Organisation study,^8^ colloquial referred to as the “10-country study” set out to report incidence rates from several settings, including LMIC contexts, data quality issues meant incidence rates were never reported for Colombia (Cali) or Nigeria (Ibadan), and published incidence data were only available for two sites in India (rural and urban Chandigarh) and Moscow in the former USSR.

1. **Detailed narrative synthesis of evidence by study quality**

All citations provided evidence of a defined catchment area and their inclusion criteria, and all but two citations^24,39^ (84·6%) reported the source of their denominator population data. Five citations (38·5%) reported use of a so-called “leakage” design,^7,8,15,22,39^ first used in the WHO 10-country study,^8^ in an attempt to identify potential cases missed by the initial case ascertainment procedure. Only one study^24^ (7·7%) reported some attempt to blind the researchers to clinical and/or demographic characteristics of the participants during the conduct of the study to reduce possible observer bias (Supplemental Table 2).

1. **Estimation of incidence provided by Ihezue (1982)^28^**

The paper by Ihezue^28^ in Anambra state, Nigeria, reported 67 new cases of schizophrenia diagnosed over a 30-day period in April 1980 in the state’s only psychiatric hospital in the state capital, Enugu. The paper reported (pp. 356) that:

“Anambra state…has a population of about 3·85 million people, based on the 1963 national census, and a projected annual growth rate of 2·5%.”

This allowed us to derive an estimated denominator for the total population and person-years at risk in April 1980. Assuming the linear growth rate continued over the 17-year period between 1963 and 1980, the estimated total population of Anambra state would be approximately:

3·85m x 1·025^17^ = 5 858 230·31 people

Assuming all people were at-risk of psychosis over the 30-day period of study, this leads to an estimated person-years at-risk of:

5 858 230.31 x (30/366) = 480 182·8 person-years

We made allowance in this calculation for the fact that 1980 was a leap year with 366 days in total. This estimate is clearly approximate, and reliant on a rounded total of the whole population reported in the paper, not all of whom would have been at-risk of schizophrenia (including children not generally considered at-risk). Nevertheless, since the reporting of the cases by Ihezue^28^ provided no restriction on age criteria, and we followed this methodology in deriving person-years at risk.

This allowed us to estimate an approximate treated incidence of schizophrenia from this study, which is highly likely to contain sources of bias in both the derivation of the true population numerator (i.e. due to a lack of a population-based case ascertainment approach) and denominator (for several reasons, including those described above).

1. **Variance by sex in incidence rates from the Jablensky et al^8^**

Sex differences in the incidence of non-affective psychotic disorders in LMICs in the WHO 10-country study varied by setting; in both India, and Moscow rates of non-affective psychotic disorders and schizophrenia were similar (urban Chandigarh, India) or higher (rural Chandigarh, India, and Moscow, in the former USSR) for women than men, although formal comparisons of these differences were not possible due to the absence of reported standard errors.

1. **Further strengths of our systematic review**

Furthermore, we searched multiple English-language databases without restrictions on place of publication or language of the original citation, and included and translated relevant citations published in a foreign language.^26,32,33^ We contacted the authors of previous systematic reviews^2^ and publishers of out-of-print works^31,32^ to minimise omitted citations which would otherwise have met inclusion criteria. Finally, citation screening at both the abstract and full text stages, and quality assessment scoring, were done in duplicate to minimise errors in identifying suitable citations. We used a previously-used quality assessment tool based on epidemiological good practice for incidence studies of psychotic disorders.^2,4^ Our comprehensive search strategy (Supplemental Materials, Section 1), and open access review database, will allow interested readers to conduct systematic reviews on specific psychotic disorder outcomes of interest, and replicate our review methodology as new data emerge.

1. **Supplemental References**

(Numbered to match references in main manuscript for consistency)

2 Kirkbride JB, Errazuriz A, Croudace TJ, et al. Incidence of schizophrenia and other psychoses in England, 1950-2009: A systematic review and meta-analyses. PLoS One 2012; 7: e31660.

3 McGrath J, Saha S, Welham J, El Saadi O, MacCauley C, Chant D. A systematic review of the incidence of schizophrenia: the distribution of rates and the influence of sex, urbanicity, migrant status and methodology. BMC Med 2004; 2: 1–22.

4 Jongsma HE, Turner C, Kirkbride JB, Jones PB. International incidence of psychotic disorders, 2002–17: a systematic review and meta-analysis. Lancet Public Heal 2019; 4: e229–44.

7 Menezes PR, Scazufca M, Busatto G, Coutinho LMS, McGuire PK, Murray RM. Incidence of first-contact psychosis in São Paulo, Brazil. Br J Psychiatry 2007; 191: 2–7.

8 Jablensky A, Sartorius N, Ernberg G, et al. Schizophrenia: manifestations, incidence and course in different cultures A World Health Organization Ten-Country Study. England, 1992 DOI:10.1017/S0264180100000904.

9 Sartorius N, Jablensky A, Korten A, et al. Early manifestations and first-contact incidence of schizophrenia in different cultures: A preliminary report on the initial evaluation phase of the WHO Collaborative Study on Determinants of Outcome of Severe Mental Disorders. Psychol Med 1986; 16: 909–28.

10 Hickling FW, Rodgers-Johnson P. The incidence of first contact schizophrenia in Jamaica. Br J Psychiatry 1995; 167: 193–6.

15 Jongsma HE, Gayer-Anderson C, Lasalvia A, et al. Treated Incidence of Psychotic Disorders in the Multinational EU-GEI Study. JAMA Psychiatry 2018; 75: 36–46.

22 Rajkumar S, Padmavathi R, Thara R, Menon MS. Incidence of schizophrenia in an urban community in Madras. Indian J Psychiatry 1993; 35: 18–21.

23 Selten J-P, Zeyl C, Dwark Asing R, Lumsden V, Kahn RS, van Harten PN. First-contact incidence of schizophrenia in Surinam. Br J Psychiatry 2005; 186: 74–5.

24 Burns JK, Esterhuizen T. Poverty, inequality and the treated incidence of first-episode psychosis. An ecological study from South Africa. Soc Psychiatry Psychiatr Epidemiol 2008; 43: 331–5.

25 Caetano R. First admission to psychiatric facilities in Brazil, 1960-1974. Bull Pan Am Health Organ 1981; 15: 148–59.

26 Handal NN, Dodds JH. Rates of first hospital admissions for schizophrenia in Costa Rica. Rev Pana Salud Publica/Pan Am J Public Heal 1997; 1: 426–34.

27 Hanoeman M, Selten J-P, Kahn RS. Incidence of schizophrenia in Surinam. Schizophr Res 2002; 54: 219–21.

28 Ihezue UH. Some observations and comments on the psychosocial profile of first‐ever referrals to the psychiatric hospital, Enugu, Nigeria. Acta Psychiatr Scand 1982; 65: 355–64.

29 Wig NN, Varma VK, Mattoo SK, et al. An incidence study of schizophrenia in India. Indian J Psychiatry 1993; 35: 11–7.

30 Tsirkin SI. [International study of schizophrenia based on a WHO program. Incidence of schizophrenia]. Zh Nevropatol Psikhiatr Im S S Korsakova 1987; 87: 1203–7.

31 Rotshtein VG. Incidence of paranoid schizophrenia. Zh Nevropatol Psikhiatr Im S S Korsakova 1982; 82: 91–8.

32 Liberman II. The incidence of schizophrenia (materials from a clinico-epidemiologic study). Zh Nevropatol Psikhiatr Im S S Korsakova 1974; 74: 1224–33.

33 Chen CH, Yucun S, Xi T, Tian Y, Wei C. Incidence and prevalence of schizophrenia in a community mental health service from 1975 to 1981. Chinese J Neurol Psychiatry 1984; 17: 321–4.

34 Shen Y, Zhang W, Shu L, et al. A survey of mental disorders in a suburb of Beijing. Int J Ment Health 1987; 16: 75–80.

35. Huang L-S, Chen Y-F, Lin X-Z, Wu Y-T (1990) A 10-year investigation on incidence of schizophrenia in community. Chin J Nerv Ment Dis 16:100–103

36. da Rocha HA, Reis IA, da Cunha Santos MA, et al (2021) Psychiatric hospitalizations by the Unified Health System in Brazil between 2000 and 2014. Rivesta de Saude Publica 55:

37. Del-Ben CM, Shuhama R, Loureiro CM, et al (2019) Urbanicity and risk of first-episode psychosis: incidence study in Brazil. The British Journal of Psychiatry 215:726–729. https://doi.org/10.1192/bjp.2019.110

38. Song J, Ramírez MC, Okano J, et al (2022) Geospatial analysis reveals distinct hotspots of severe mental illness. medRxiv 2022.03.23.22272776. https://doi.org/10.1101/2022.03.23.22272776

39 Morgan C, John S, Esan O, et al. The incidence of psychoses in diverse settings, INTREPID (2): a feasibility study in India, Nigeria, and Trinidad. Psychol Med 2016; 46: 1923–33.

**Supplemental Figure 1: Correlation between study quality and mid-year of case ascertainment ^a^**

^a^ Based on 16 out of 18 core citations for which a study quality rating could be made from the full text. Mid-point of case ascertainment is displayed on the x-axis, with each citation labelled by year of publication, consistent with our reporting of these citations in the tables. Maximum study quality score was seven (see Supplemental Table 2 for details of quality scores)

**Supplemental Figure 2: Funnel plot of incidence rates of non-affective psychotic disorders from LMICs by sample size (standard error)**


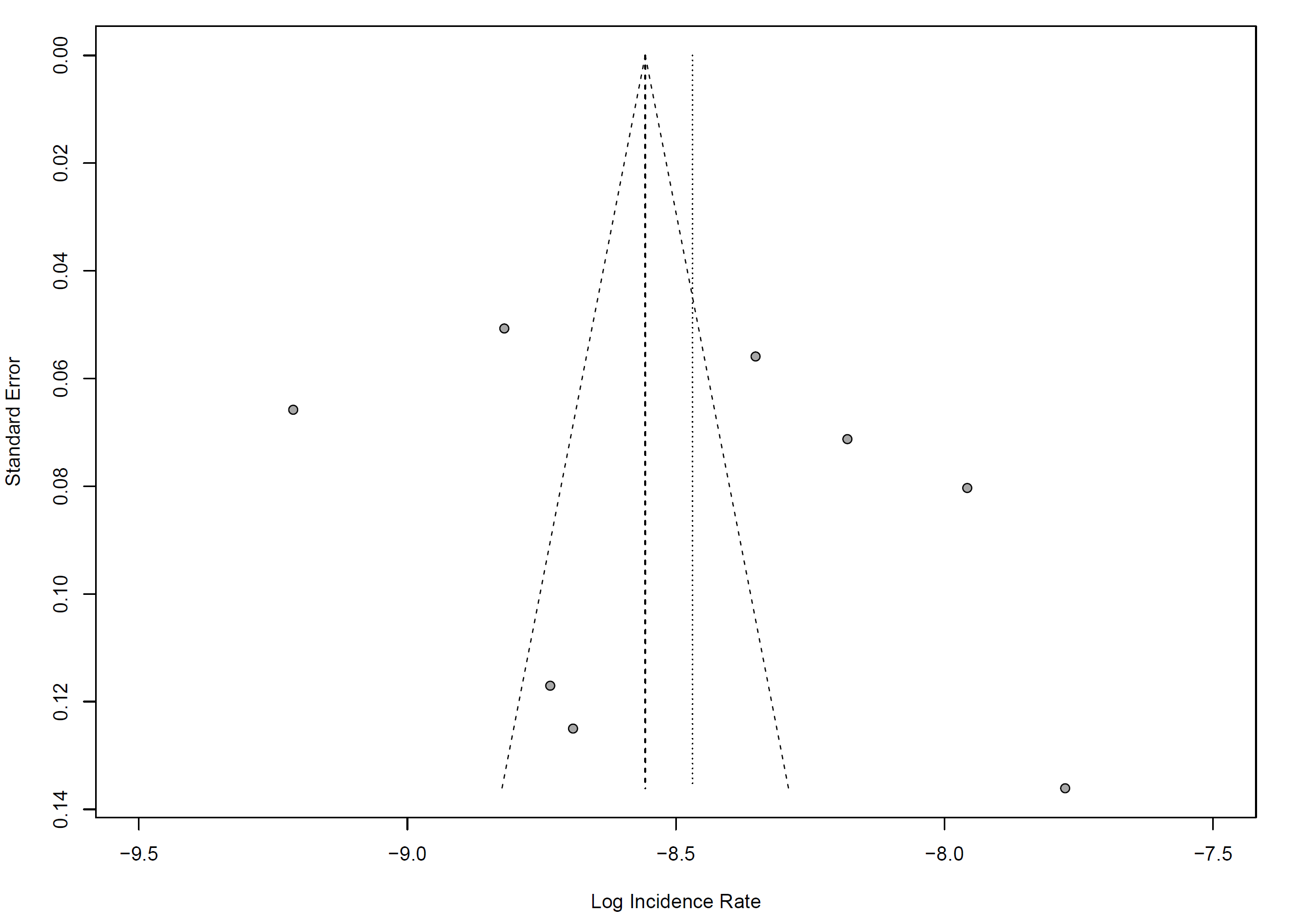


**Supplemental Figure 3: Funnel plot of incidence rates of schizophrenia from LMICs by sample size (standard error)^a^**


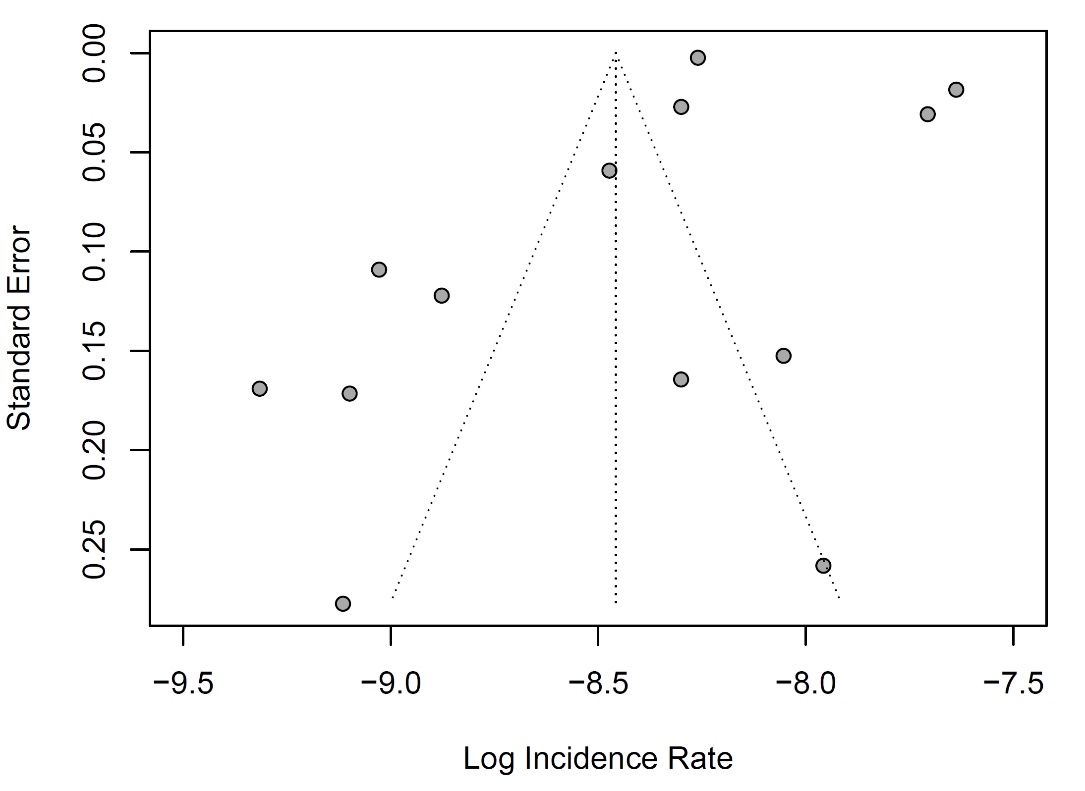


^a^ We performed an Egger’s test for small study effects on the incidence rates of schizophrenia (N=13), which provided no evidence of funnel plot asymmetry (p=0·70).

**Supplemental Figure 4: Funnel plot of incidence rates of all clinically-relevant psychotic disorders from LMICs by sample size (standard error)**


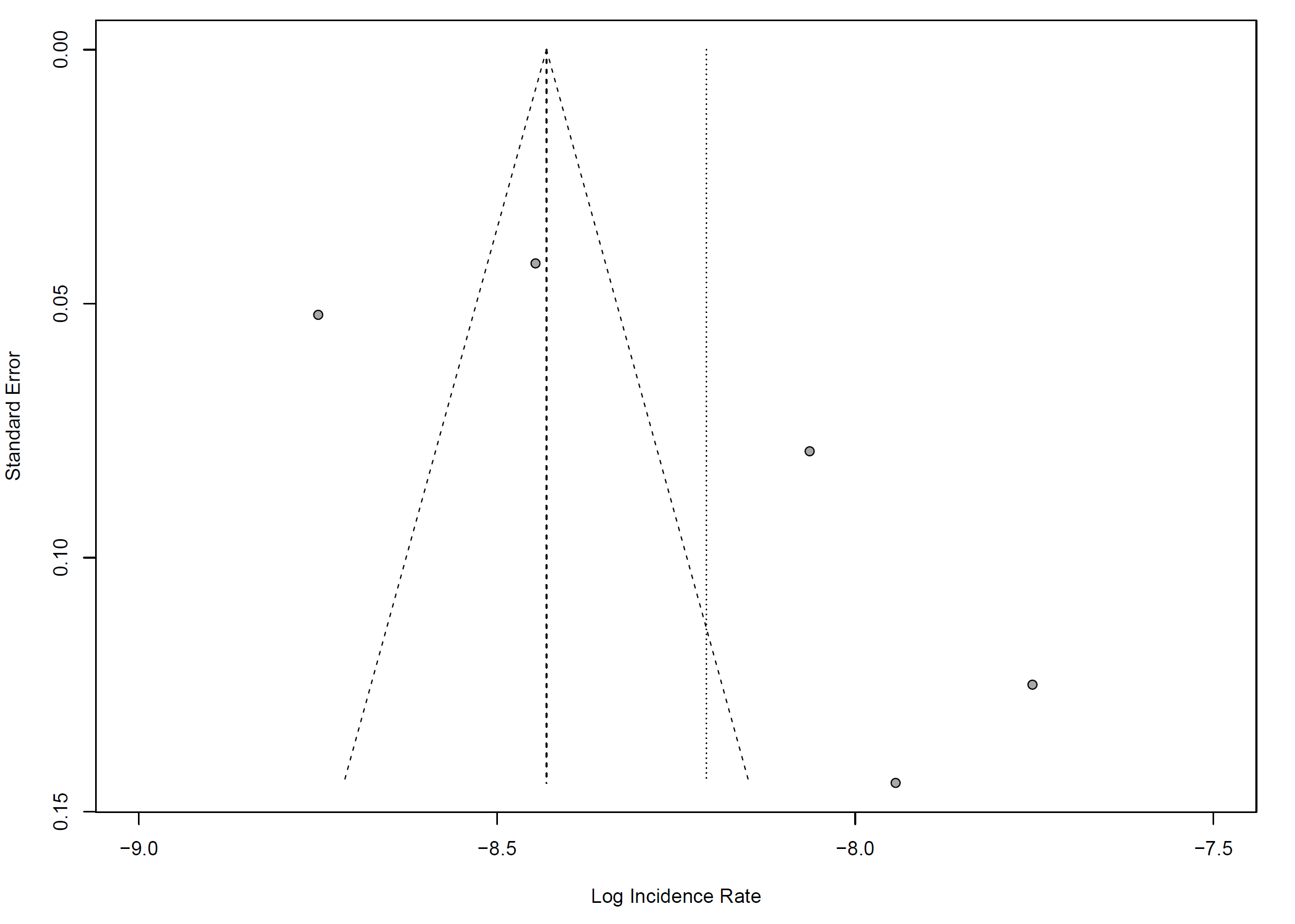


**Supplemental Table 1: PRISMA Statement**

| **Section/topic** | **#** |  | **Reported on page #** | |  |
| --- | --- | --- | --- | --- | --- |
| **TITLE** | | | |  | |
| Title | 1 | Identify the report as a systematic review, meta-analysis, or both. | 1 | |  |
| **ABSTRACT** | | | |  | |
| Structured summary | 2 | Provide a structured summary including, as applicable: background; objectives; data sources; study eligibility criteria, participants, and interventions; study appraisal and synthesis methods; results; limitations; conclusions and implications of key findings; systematic review registration number. | 2 | |  |
| **INTRODUCTION** | | | |  | |
| Rationale | 3 | Describe the rationale for the review in the context of what is already known. | 4 | |  |
| Objectives | 4 | Provide an explicit statement of questions being addressed with reference to participants, interventions, comparisons, outcomes, and study design (PICOS). | 4 | |  |
| **METHODS** | | | |  | |
| Protocol and registration | 5 | Indicate if a review protocol exists, if and where it can be accessed (e.g., Web address), and, if available, provide registration information including registration number. | 5 | |  |
| Eligibility criteria | 6 | Specify study characteristics (e.g., PICOS, length of follow-up) and report characteristics (e.g., years considered, language, publication status) used as criteria for eligibility, giving rationale. | 5 | |  |
| Information sources | 7 | Describe all information sources (e.g., databases with dates of coverage, contact with study authors to identify additional studies) in the search and date last searched. | 5 | |  |
| Search | 8 | Present full electronic search strategy for at least one database, including any limits used, such that it could be repeated. | Supp. Section 1 | |  |
| Study selection | 9 | State the process for selecting studies (i.e., screening, eligibility, included in systematic review, and, if applicable, included in the meta-analysis). | 5 | |  |
| **Section/topic** | **#** | **Checklist item** | **Reported on page #** | |  |
| Data collection process | 10 | Describe method of data extraction from reports (e.g., piloted forms, independently, in duplicate) and any processes for obtaining and confirming data from investigators. | 5-6 | |  |
| Data items | 11 | List and define all variables for which data were sought (e.g., PICOS, funding sources) and any assumptions and simplifications made. | 5-6, Supp. Sections 3-4 | |  |
| Risk of bias in individual studies | 12 | Describe methods used for assessing risk of bias of individual studies (including specification of whether this was done at the study or outcome level), and how this information is to be used in any data synthesis. | 6, Supp. Section 4 | |  |
| Summary measures | 13 | State the principal summary measures (e.g., risk ratio, difference in means). | 6 | |  |
| Synthesis of results | 14 | Describe the methods of handling data and combining results of studies, if done, including measures of consistency (e.g., I^2^) for each meta-analysis. | 6 | |  |
| Risk of bias across studies | 15 | Specify any assessment of risk of bias that may affect the cumulative evidence (e.g., publication bias, selective reporting within studies). | 6 | |  |
| Additional analyses | 16 | Describe methods of additional analyses (e.g., sensitivity or subgroup analyses, meta-regression), if done, indicating which were pre-specified. | N/A | |  |
| **RESULTS** | | | |  | |
| Study selection | 17 | Give numbers of studies screened, assessed for eligibility, and included in the review, with reasons for exclusions at each stage, ideally with a flow diagram. | 7, Figure 1 | |  |
| Study characteristics | 18 | For each study, present characteristics for which data were extracted (e.g., study size, PICOS, follow-up period) and provide the citations. | 7, Table 1, Supp. Table 2 | |  |
| Risk of bias within studies | 19 | Present data on risk of bias of each study and, if available, any outcome level assessment (see item 12). | 7-10 | |  |
| Results of individual studies | 20 | For all outcomes considered (benefits or harms), present, for each study: (a) simple summary data for each intervention group (b) effect estimates and confidence intervals, ideally with a forest plot. | 7-10, Figure 2 | |  |
| Synthesis of results | 21 | Present results of each meta-analysis done, including confidence intervals and measures of consistency. | 7-8, Figure 2 | |  |

| **Section/topic** | **#** | **Checklist item** | **Reported on page #** | |  |
| --- | --- | --- | --- | --- | --- |
| Risk of bias across studies | 22 | Present results of any assessment of risk of bias across studies (see Item 15). | 10, Supp. Section 6, Supp. Table 2, Supp. Figures 1-4 | |  |
| Additional analysis | 23 | Give results of additional analyses, if done (e.g., sensitivity or subgroup analyses, meta-regression [see Item 16]). | N/A | |  |
| **DISCUSSION** | | | |  | |
| Summary of evidence | 24 | Summarize the main findings including the strength of evidence for each main outcome; consider their relevance to key groups (e.g., healthcare providers, users, and policy makers). | 11 | |  |
| Limitations | 25 | Discuss limitations at study and outcome level (e.g., risk of bias), and at review-level (e.g., incomplete retrieval of identified research, reporting bias). | 11-12 | |  |
| Conclusions | 26 | Provide a general interpretation of the results in the context of other evidence, and implications for future research. | 12-13 | |  |
| **FUNDING** | | | |  | |
| Funding | 27 | Describe sources of funding for the systematic review and other support (e.g., supply of data); role of funders for the systematic review. | 14 | |  |

*From:*  Moher D, Liberati A, Tetzlaff J, Altman DG, The PRISMA Group (2009). Preferred Reporting Items for Systematic Reviews and Meta-Analyses: The PRISMA Statement. PLoS Med 6(7): e1000097. doi:10.1371/journal.pmed1000097

For more information, visit: **www.prisma-statement.org**.

**Supplemental Table 2 Quality appraisal of included citations**

| **First author (year)** | **Core citation** | **Defined catchment** | **Accurate denominator** | **Population-based case finding** | **Standardised diagnoses** | **Blinding to demographic variables** | **Inclusion criteria clear** | **Leakage study** | **Quality Score** |
| --- | --- | --- | --- | --- | --- | --- | --- | --- | --- |
| Burns (2008)^24^ | Yes | 1 | 0 | 1 | 0 | 1 | 1 | 0 | 4 |
| Caetano (1981)^25^ | Yes | 1 | 1 | 0 | 0 | 0 | 0·5 | 0 | 2·5 |
| Chen (1984)^33^ | Yes | 1 | 1 | 0 | 1 | 0 | 0 | 0 | 3 |
| Da Rocha (2021)^36^ | Yes | 1 | 1 | 0 | 0 | 0 | 1 | 0 | 3 |
| Handal (1997)^26^ | Yes | 1 | 1 | 0 | 0 | 0 | 1 | 0 | 3 |
| Hanoeman (2002)^27^ | Yes | 1 | 1 | 1 | 0 | 0 | 1 | 0 | 4 |
| Hickling (1995)^10^ | Yes | 1 | 1 | 0 | 1 | 0 | 0·5 | 0 | 3·5 |
| Huang (1990)^35^ | Yes | 1 | 1 | 0 | 1 | 0 | 0 | 0 | 3 |
| Ilhezue (1982)^28^ | Yes | 1 | 0 | 0 | 0 | 0 | 0 | 0 | 1 |
| Jablensky (1992)^8^ | Yes | 1 | 1 | 1 | 1 | 0 | 1 | 1 | 6 |
| Jongsma (2018)^15^ | Yes | 1 | 1 | 1 | 1 | 0 | 1 | 1 | 6 |
| Liberman (1974)^32^ | Yes | - | - | - | - | - | - | - | NR^a^ |
| Menezes (2007)^7^ | Yes | 1 | 1 | 1 | 1 | 0 | 1 | 1 | 6 |
| Morgan (2016)^39^ | Yes | 1 | 0 | 1 | 1 | 0 | 1 | 1 | 5 |
| Rajkumar (1993)^22^ | Yes | 1 | 1 | 1 | 1 | 0 | 1 | 1 | 6 |
| Rotshteĭn (1982)^31^ | Yes | - | - | - | - | - | - | - | NR^a^ |
| Sartorius (1986)^9^ | No^b^ | 1 | 1 | 1 | 1 | 0 | 1 | 1 | 6 |
| Selten (2005)^23^ | Yes | 1 | 1 | 1 | 0 | 0 | 1 | 0 | 4 |
| Shen (1987)^34^ | No^c^ | 1 | 0 | 1 | 0 | 0 | 0 | 0 | 2 |
| Song (2022)^38^ | Yes | 1 | 0 | 0 | 0 | 0 | 1 | 0 | 2 |
| Tsirkin (1987)^30^ | No^b^ | - | - | - | - | - | - | - | NR^a^ |
| Wig (1993)^29^ | No^b^ | 1 | 1 | 1 | 1 | 0 | 1 | 1 | 6 |
| **Total (N=16)^d^** |  | 16 | 13 | 8 | 8 | 1 | 12 | 5 | - |
| **% of core citations^d^** |  | 100·0 | 81·3 | 50·0 | 50·0 | 6·3 | 75·0 | 31·3 |  |

^a^ Full text unavailable so ratings could not be performed

^b^ Duplicate citations of the WHO 10-country study – see Jablensky (1992)

^c^ Duplication citation of Chen et al (1984)

^d^ Sixteen core citations, for which full texts were available, could be assigned a quality score
